# Estrogen receptor-dependent mechanisms underlying DES promoted endometrial carcinoma: Study in vivo

**DOI:** 10.1016/j.eurox.2026.100504

**Published:** 2026-10-17

**Authors:** Setyo Teguh Waluyo, Brahmana Askandar Tjopkroprawiro, Peng-Hui Wang, Widjiati Widjiati

**Affiliations:** a Doctoral Program of Medical Science, Medical Faculty, Universitas Airlangga, Surabaya, Indonesia; b Department of Obstetrics and Gynecology, Medical Faculty, Universitas Airlangga, Surabaya, Indonesia; c Department of Obstetrics and Gynecology, Dr. Soetomo General Academic Hospital, Surabaya, Indonesia; d Department of Obstetrics and Gynecology, Taipei Veterans General Hospital, Taipei, Taiwan; e Department of Veterinary Medical Science, Faculty of Veterinary Medicine, Universitas Airlangga, Surabaya, Indonesia

**Keywords:** Diethylstilbestrol, Estrogen receptor alpha, Endometrial carcinogenesis, Endocrine disrupting chemicals, In vivo study, Immunohistochemistry

## Abstract

**Background:**

Diethylstilbestrol (DES) is a potent synthetic estrogen implicated in endometrial carcinogenesis through estrogen receptor (ER)-dependent mechanisms. However, the temporal relationship between DES exposure, estrogen receptor alpha (ERα) expression, and histopathological progression of endometrial lesions remains incompletely defined in vivo.

**Methods:**

An experimental in vivo study was conducted using 32 eight-week-old female mice randomly assigned to eight groups, including two untreated controls and six DES-treated groups with varying exposure and observation durations. DES was administered at 1000 µg according to predefined schedules. Uterine tissues were examined histopathologically using HE staining, and ERα expression was evaluated by immunohistochemistry using the modified Remmele Immunoreactive Score. Statistical analyses included one-way ANOVA with Tukey post hoc testing, Kruskal–Wallis and Mann–Whitney U tests, and path analysis to assess mechanistic relationships.

**Results:**

ERα expression differed significantly among groups, with the highest expression observed in mice receiving prolonged DES exposure followed by extended observation. Histopathological evaluation demonstrated a clear progression from normal endometrium in control groups to higher-grade lesions in DES-treated groups, with severe (Grade 3) changes predominating in high exposure cohorts. Path analysis revealed a significant association between DES exposure and ERα expression, as well as a positive effect of ERα on EC development.

**Conclusions:**

DES promotes endometrial carcinogenesis through ERα dependent mechanisms in vivo. Increased ERα expression parallels histopathological progression, and mechanistic analysis confirms ERα as a key mediator linking synthetic estrogen exposure to tumor development. These findings underscore the central role of estrogen signaling in DES-induced EC.

## Highlights

• DES exposure significantly increases ERα expression in endometrial tissue.
• Prolonged DES treatment leads to progressive endometrial lesion severity.
• ERα upregulation is associated with histopathological tumor progression.
• Findings highlight estrogen signaling as a key driver in DES-related EC.

## Introduction

Diethylstilbestrol (DES), a synthetic estrogen, has been extensively studied due to its role as a carcinogen, particularly in the context of endometrial carcinoma (EC). The effects of DES are mediated through estrogen receptor (ER) pathways, which play critical roles in the differentiation, proliferation, and transformation of endometrial cells [1].

Studies have shown that estrogen and its receptors are pivotal in the development and progression of EC. For instance, Kabbarah et al. established that DES treatment of mice resulted in distinct gene expression changes associated with estrogen's tumor-promoting effects, emphasizing the importance of ER pathways in endometrial carcinogenesis [2].

Moreover, a direct relationship between ER expression levels and histopathological progression from normal endometrium to hyperplasia and ultimately carcinogenesis has been demonstrated Guo et al., the complexity of these changes suggests that both ERα and ERβ may influence different aspects of endometrial tumor behavior, contributing to the diverse pathological states observed in human endometrial neoplasms [3].

The hormonal environment during critical periods of development has been implicated in modifying the risk of EC. Evidence indicates that estrogen, when not counterbalanced by progesterone, induces a carcinogenic environment. This has been illustrated in studies showing that high estrogen levels significantly correlate with increased EC risk and tumor aggressiveness [3], [4]. Furthermore, DES's impact on the endometrial microenvironment, such as upregulating matrix metalloproteinases and promoting angiogenesis, underscores the multifaceted nature of DES-induced carcinogenesis [5].

The present study delves into the intricate link between ER-dependent mechanisms and the carcinogenic effects of DES in the endometrium, providing insights into both the biological pathways involved and the broader implications for clinical management of EC. By elucidating how ER expression impacts DES-mediated endometrial carcinogenesis, we hope to augment the understanding of this disease and foster the development of targeted prevention and treatment strategies.

## Materials and methods

### Ethics statement

The present study was approved by the ethics committee, in accordance with the ethical guidelines for animal studies established by the Faculty of Veterinary Medicine, Universitas Airlangga Surabaya, Indonesia (Approval Num. 2.KEH.69.04.2026). The animal research was carried out with strict adherence to the accepted standards for the care and use of laboratory animals of Faculty of Veterinary Medicine, Universitas Airlangga. The protocol was approved by the ethics committee, in accordance with the ethical guidelines for animal studies established by the Faculty of Veterinary Medicine, Universitas Airlangga. All efforts were made to minimize animal suffering.

### Animals and experimental design

All experimental procedures were conducted in accordance with institutional guidelines for the care and use of laboratory animals. This experimental study used 8-week-old female mice. A total of 32 mice were included and randomly assigned into eight experimental groups, with four animals per group. Two groups served as untreated controls. The first control group consisted of mice observed for 10 days without any intervention, while the second control group consisted of mice observed for 15 days without any intervention. The remaining six groups constituted the treatment groups and received DES at a dose of 1000 µg according to different treatment and observation schedules (Fig. 1).

**Fig. 1:**
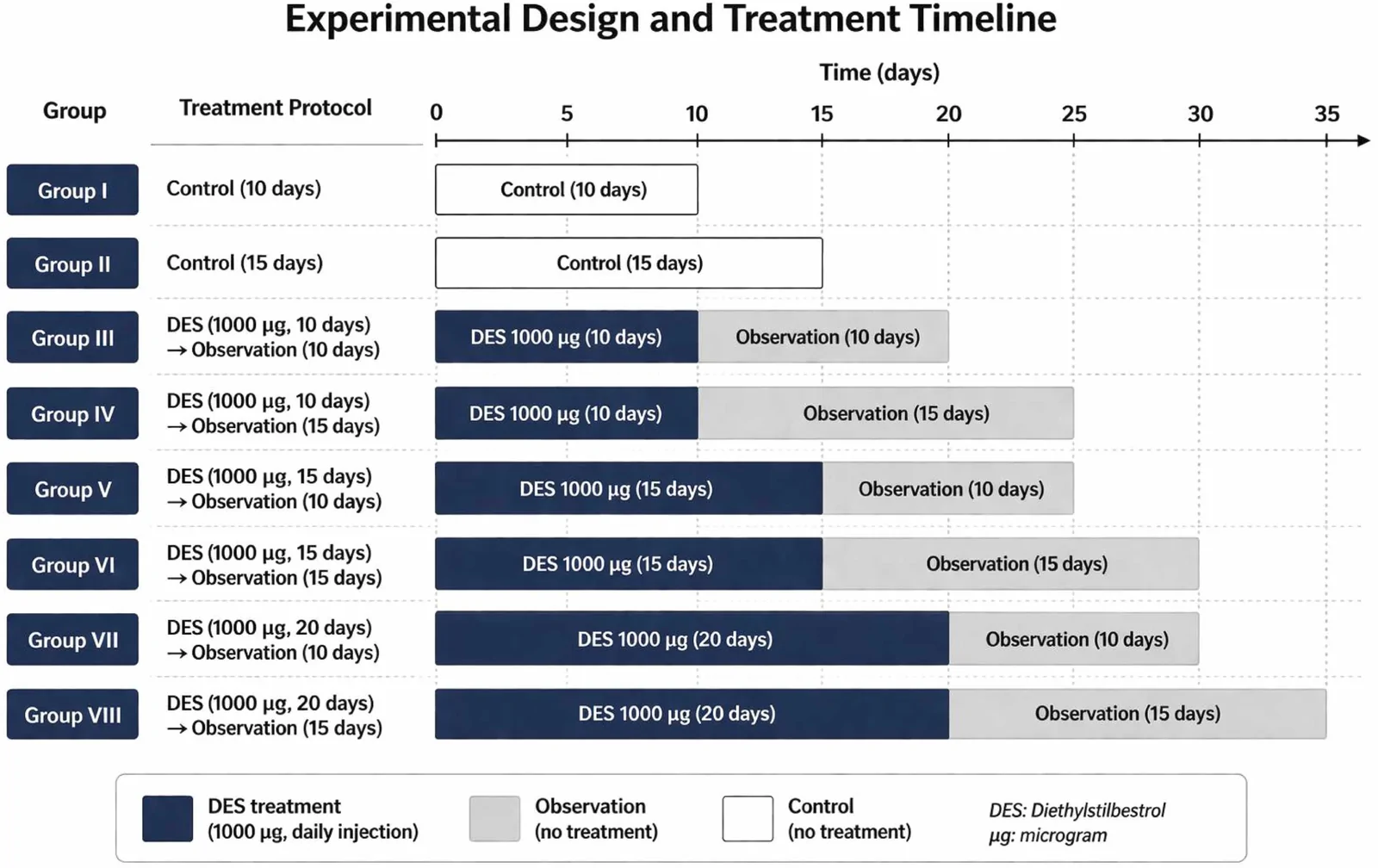
Schematic timeline of the experimental design.

The selected dose of 1000 µg DES was based on established experimental models of estrogen-induced carcinogenesis. Previous study has demonstrated that neonatal or early-life exposure to DES at approximately 1000 µg/kg/day induces a high incidence of uterine adenocarcinoma in rodents, with more than 90% of exposed animals developing tumors later in life. These tumors follow a classical hormonal carcinogenesis model characterized by an initiation–promotion mechanism, in which early developmental exposure acts as the initiating event, while subsequent ovarian hormone exposure during puberty serves as a promoting factor [6].

### Tissue collection and histological processing

At their respective experimental endpoints, mice were euthanized by carbon dioxide (CO₂) asphyxiation in accordance with institutional animal care guidelines. The uterine were immediately harvested. The remaining female reproductive tract tissues were fixed in 10% neutral buffered formalin, processed using standard histological procedures, and sectioned longitudinally at a thickness of 6 µm. Tissue sections were stained with hematoxylin and eosin (HE) for histopathological evaluation or left unstained for subsequent immunohistochemical analysis.

### Histopathologic analysis

One representative HE stained uterine section from each mouse was examined using light microscopy. Histopathological grading of endometrial lesions was performed according to the International Federation of Gynecology and Obstetrics (FIGO) classification system for endometrial carcinoma (Table 1) [7].

**Table 1:** FIGO grading system for endometrial carcinoma [7].

All histopathologic sections were reviewed for tissue integrity and proper orientation prior to analysis. Histopathologic assessments were independently performed by an experienced veterinary anatomic pathologist who was blinded to treatment group allocation during evaluation. Microscopic examination was conducted using a Nikon Eclipse Ci-E light microscope. Representative photomicrographs were acquired using a Nikon DS-Fi2 high-resolution digital camera and processed with Nikon Image System software.

### Immunohistochemical analysis

Mouse uterine sections were immunostained for ERα antibody. The overall abundance of ERα immunolabeling was evaluated by light microscopy and assessed semi-quantitatively using an ERα expression scoring system according to the modified Remmele method (Table 2) [8].

**Table 2:** Immunoreactive score (IRS) scoring system [8].

For each sample, the final IRS value was expressed as the mean score obtained from five different high-power fields (HPFs) examined at 400 × magnification. All examinations were performed using a Nikon H600L light microscope equipped with a DS-Fi2 digital camera and Nikon Image System software for image acquisition and analysis.

### Statistical analysis

The sample size was calculated based on the formula for estimation of population parameters for continuous outcomes with independent group comparisons [9]. The primary outcome in this study was continuous data, and comparisons were performed between independent groups receiving different treatments.

The sample size was calculated using the following formula: n = 2 × SD² × (Zα/2 + Zβ)² / d² where SD represents the standard deviation obtained from previous study (0.6), Zα/2 is the standard normal deviate corresponding to a two-sided type I error of 5% (1.96), Zβ corresponds to a statistical power of 80% (0.84), and d represents the expected difference in means (1.27) [10].

Based on this calculation, the minimum required sample size per group was 3.499, which was rounded up to four animals per group. To account for a potential dropout rate of approximately 30%, an adjusted sample size of up to six animals per group was considered. However, the final sample size used in this study was four animals per group, in accordance with ethical considerations to minimize animal use while maintaining sufficient statistical power.

Statistical analysis were performed to evaluate differences in ERα expression and histopathological result among experimental groups and to explore the structural relationships between DES exposure, ERα expression, and EC outcomes. Parametric and non-parametric tests were applied according to data characteristics. Continuous variables (ER expression scores) that met assumptions of normality and homogeneity were analyzed using parametric tests, whereas ordinal variables (histopathological grades) were analyzed using non-parametric tests.

Normality of ERα score distributions within each group was assessed using the Shapiro–Wilk test, and homogeneity of variances was evaluated with Levene’s test. Because assumptions of normality and variance homogeneity were satisfied, one-way analysis of variance (ANOVA) was applied to compare mean ERα scores across groups. When overall group differences were significant, Tukey’s honestly significant difference (HSD) test was used for post hoc pairwise comparisons to control for type I error due to multiple testing. A p-value < 0.05 was considered statistically significant.

Histopathological results, treated as ordinal variables, were analyzed using the Kruskal–Wallis test to assess overall differences among groups. Significant results were followed by pairwise Mann–Whitney U tests to identify intergroup differences, with two-tailed p values reported.

To investigate potential mechanistic relationships between DES exposure, ERα expression, and EC development, path analysis was conducted. Standardized path coefficients (β), t statistics, and p values were calculated to estimate direct effects. Model explanatory power was evaluated using R² values for endogenous variables. All statistical tests were two-sided, and a p value < 0.05 was considered statistically significant.

## Results

### Estrogen receptor alpha expression

A total of 32 specimens distributed across eight groups (K1, K2, P1.1, P1.2, P2.1, P2.2, P3.1, and P3.2; n = 4 per group) were analyzed for ERα expression. Descriptive analysis demonstrated substantial intergroup variability in ERα scores, with mean values ranging from 0.85 ± 0.72 in group P3.1–5.40 ± 2.26 in group P2.2. Control groups (K1 and K2) showed moderate ERα expression (means: 3.60 ± 2.08 and 2.50 ± 1.55, respectively), whereas treatment groups exposed to higher experimental intensity exhibited markedly elevated receptor expression, particularly P2.2 and P3.2 (Fig. 2, Fig. 3).

**Fig. 2:**
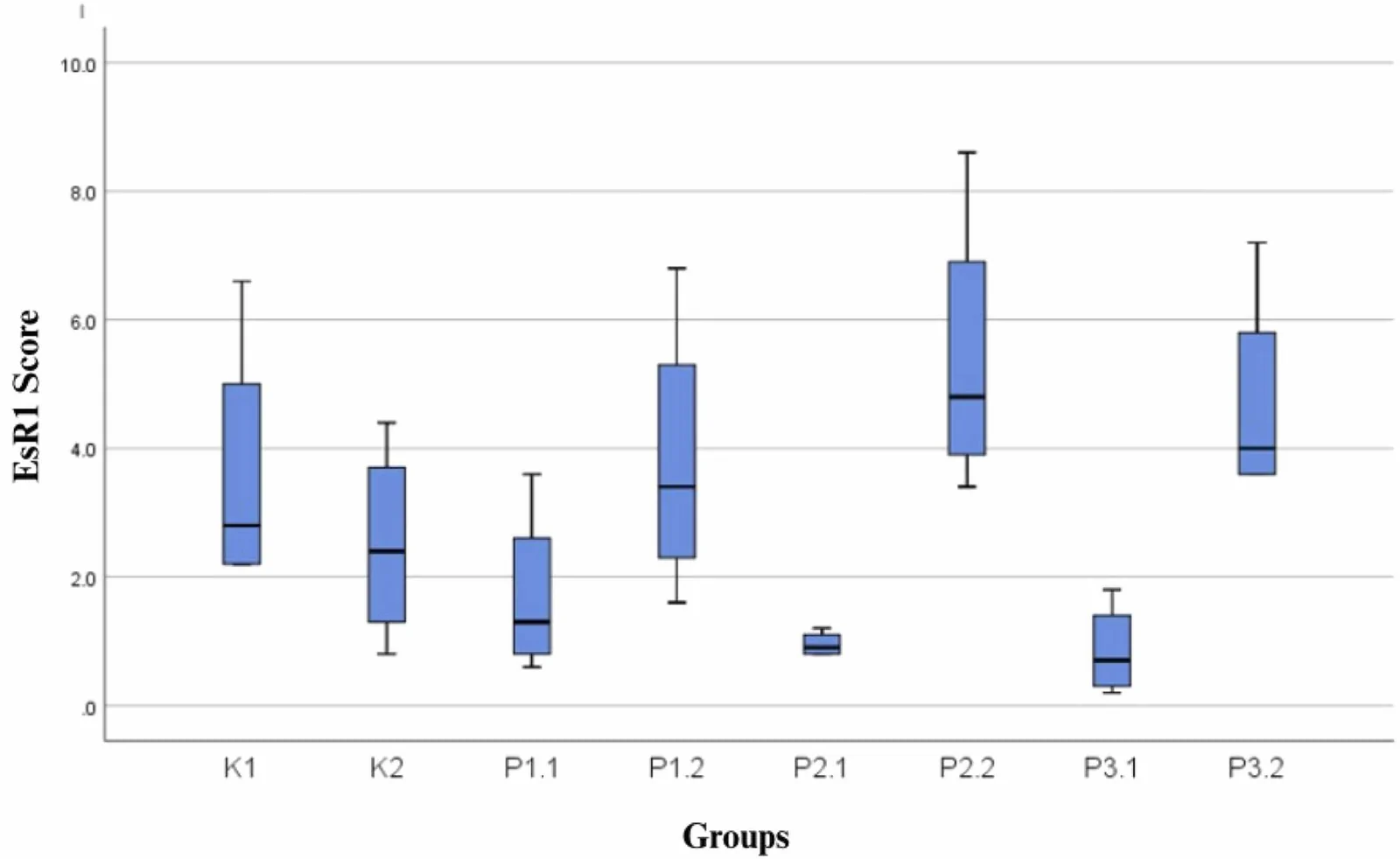
Distribution of EsR1/ ERα expression.

**Fig. 3:**
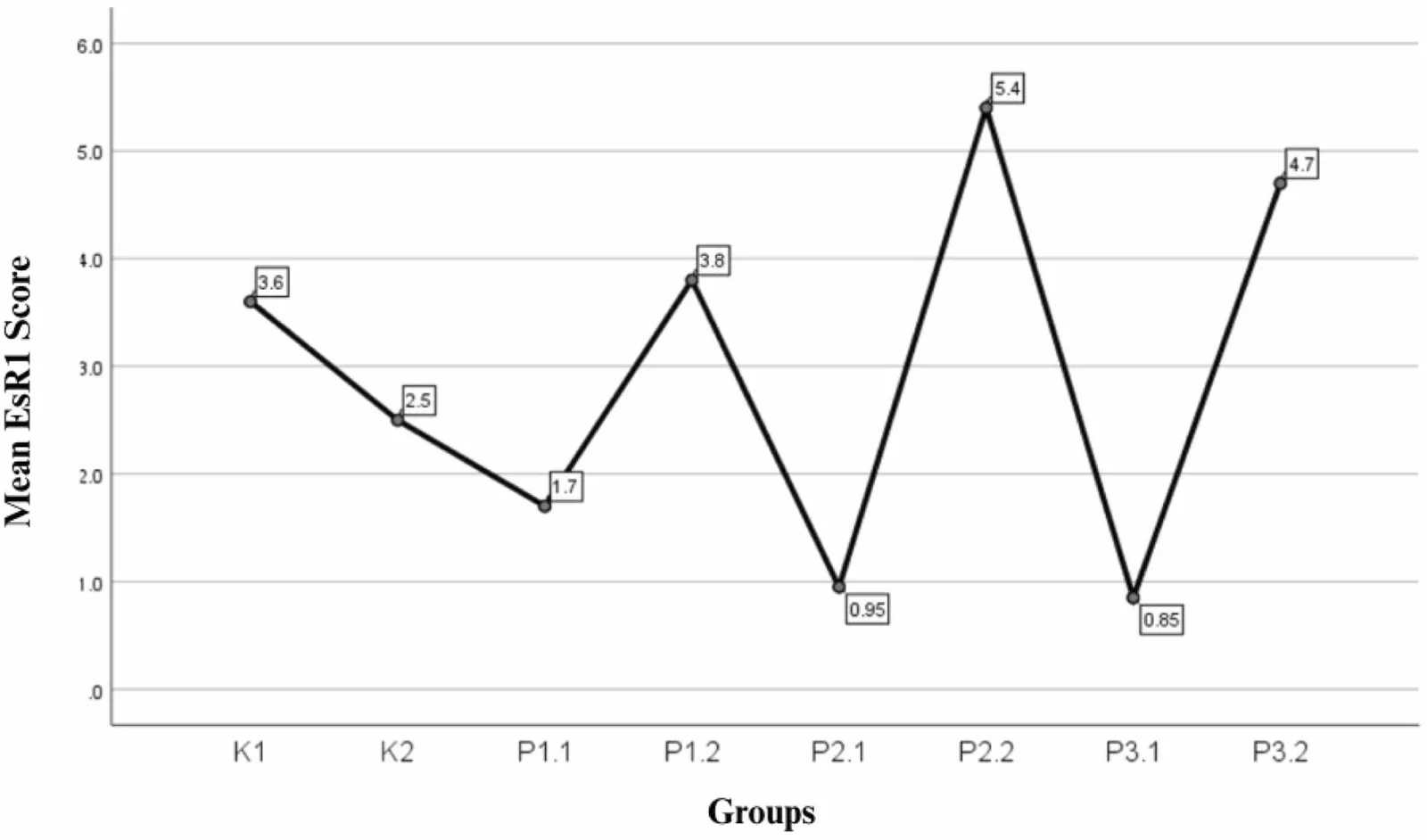
Distribution of mean of EsR1/ ERα expression score.

Normality testing using the Shapiro–Wilk method indicated no significant deviation from normal distribution in any group (p > 0.05). Homogeneity of variance was confirmed by Levene’s test (p = 0.330), permitting parametric comparison. One-way ANOVA revealed a statistically significant difference in ERα expression among groups (F = 4.255, p = 0.003). Post hoc analysis using Tukey’s HSD test was performed to account for multiple comparisons. Most group comparisons were not statistically significant and formed overlapping homogeneous subsets. However, group P2.2 demonstrated significantly higher ERα expression compared with P2.1 (mean difference = 4.45, p = 0.017) and P3.1 (mean difference = 4.55, p = 0.014), while other comparisons were not statistically significant. These findings indicate that ERα expression is associated with both exposure duration and observation period.

### Histopathological result and grade

Histopathological evaluation revealed a clear progression of endometrial abnormalities across experimental groups. Control groups (K1 and K2) predominantly exhibited normal histology (75%) with occasional Grade 1 changes (25%). In contrast, treatment groups showed increased frequencies of higher grade lesions. Group P2.2 demonstrated the most severe alterations, with 75% of specimens classified as Grade 3, while P1.2 and P3.2 showed predominance of Grade 2 lesions (75% and 50%, respectively) (Fig. 4).

**Fig. 4:**
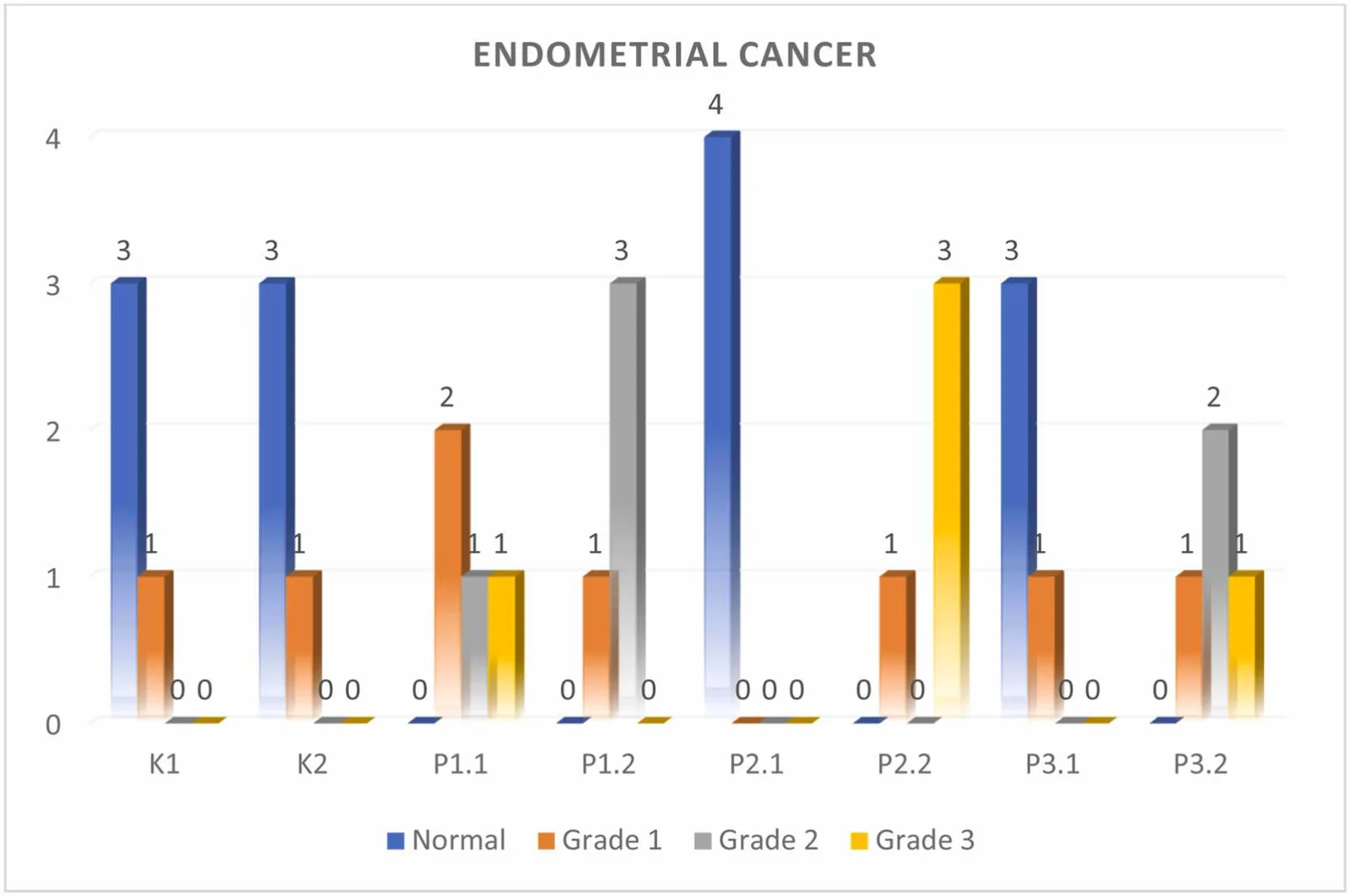
Distribution of histopathological result.

The Kruskal–Wallis test confirmed a significant difference in histopathological grades among groups (H = 23.604, p = 0.001). Pairwise Mann–Whitney analyses showed that several treatment groups (P1.1, P1.2, P2.2, and P3.2) differed significantly from controls (K1 and K2), supporting a treatment-associated escalation of pathological severity.

### Path analysis of DES exposure, ERα expression, and EC

Path analysis was conducted to evaluate the direct and indirect effects of diethylstilbestrol (DES) exposure on endometrial cancer, with estrogen receptor 1 (EsR1) as a potential mediator. The model demonstrated significant pathways between the variables (Fig. 5). DES exposure showed a significant positive effect on EsR1 expression (β = 0.384, p = 0.002), indicating that DES upregulates estrogen receptor signaling. In turn, EsR1 expression was significantly associated with an increased risk of endometrial cancer (β = 0.482, p = 0.001), suggesting its critical role in tumor development. Additionally, DES exhibited a direct effect on endometrial cancer (β = 0.232), indicating that DES contributes to carcinogenesis through both receptor-dependent and receptor-independent mechanisms.

**Fig. 5:**
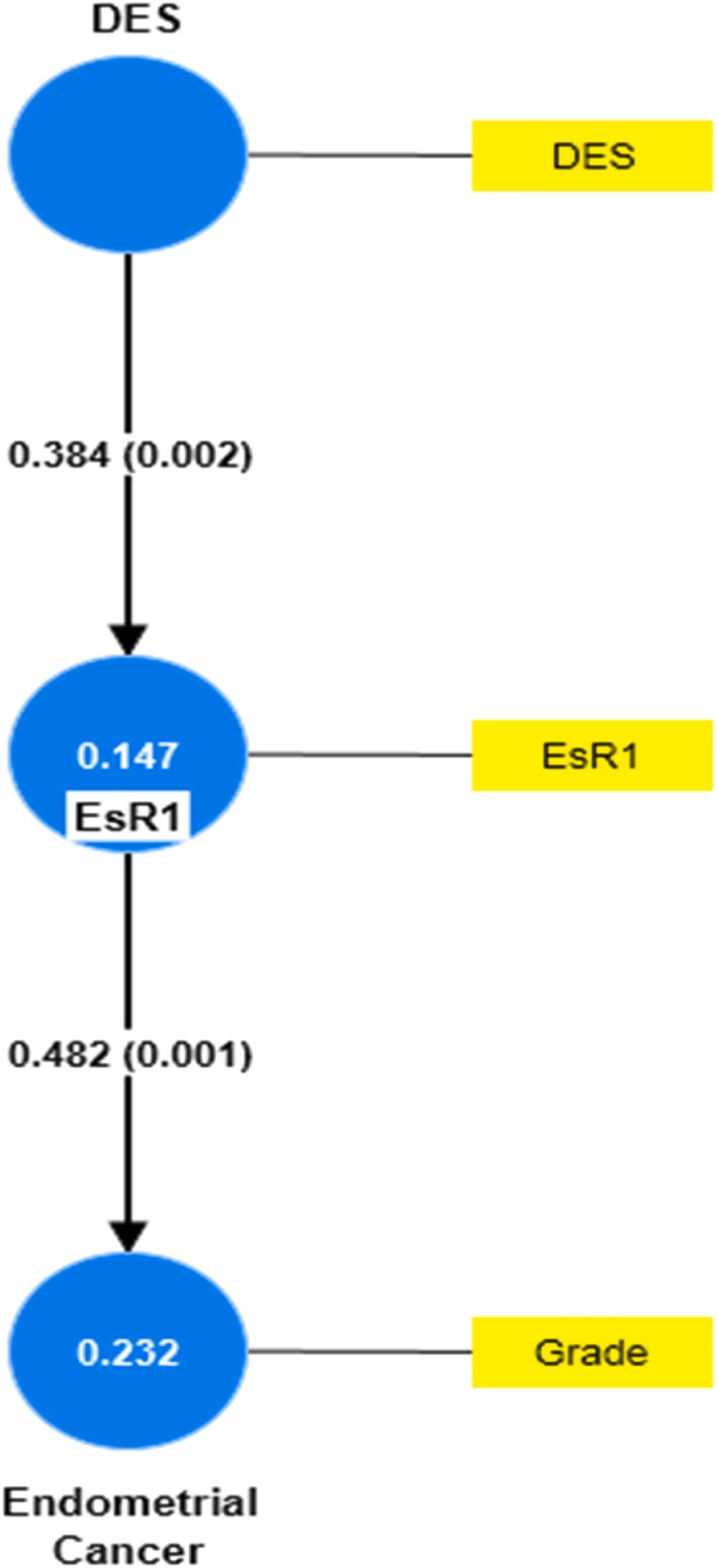
Path analysis of DES exposure, ERα expression, and EC.

The calculated indirect effect of DES on endometrial cancer via EsR1 was 0.185, reflecting a substantial mediating role of EsR1 in this relationship. These findings indicate that EsR1 partially mediates the effect of DES on endometrial cancer development, with both direct and indirect pathways contributing to the overall effect.

Macroscopic examination showed differences in uterine morphology between the control and DES-treated groups (Fig. 6). The uterus from control group appeared grossly unremarkable while the P2.2 group showed an enlarged uterus with hyperemic mucosal and serosal surfaces, and the presence of a pathological mass is observed.

**Fig. 6:**
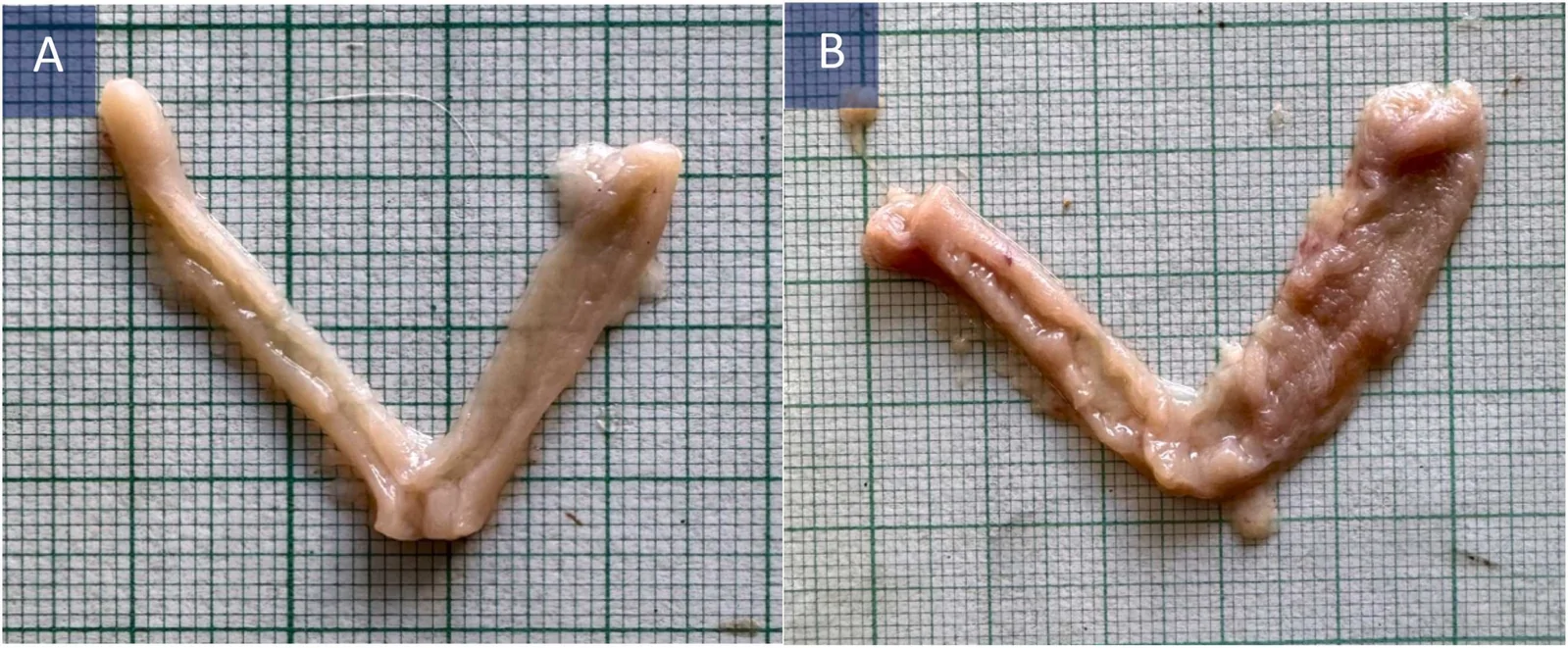
Macroscopic alterations of mouse uterine tissue following DES administration. (A) Uterine tissue from the control group. (B) Uterine tissue from the group treated with DES for 15 consecutive days and followed up for an additional 15 days (P2.2).

Histopathological examination of uterine tissue from DES-treated mice demonstrated various epithelial and glandular abnormalities. These included atypical endometrial epithelial proliferation, nuclear polarity, heterogeneous epithelial morphology, and areas with villoglandular, papillary, and mucinous features (Fig. 7).

**Fig. 7:**
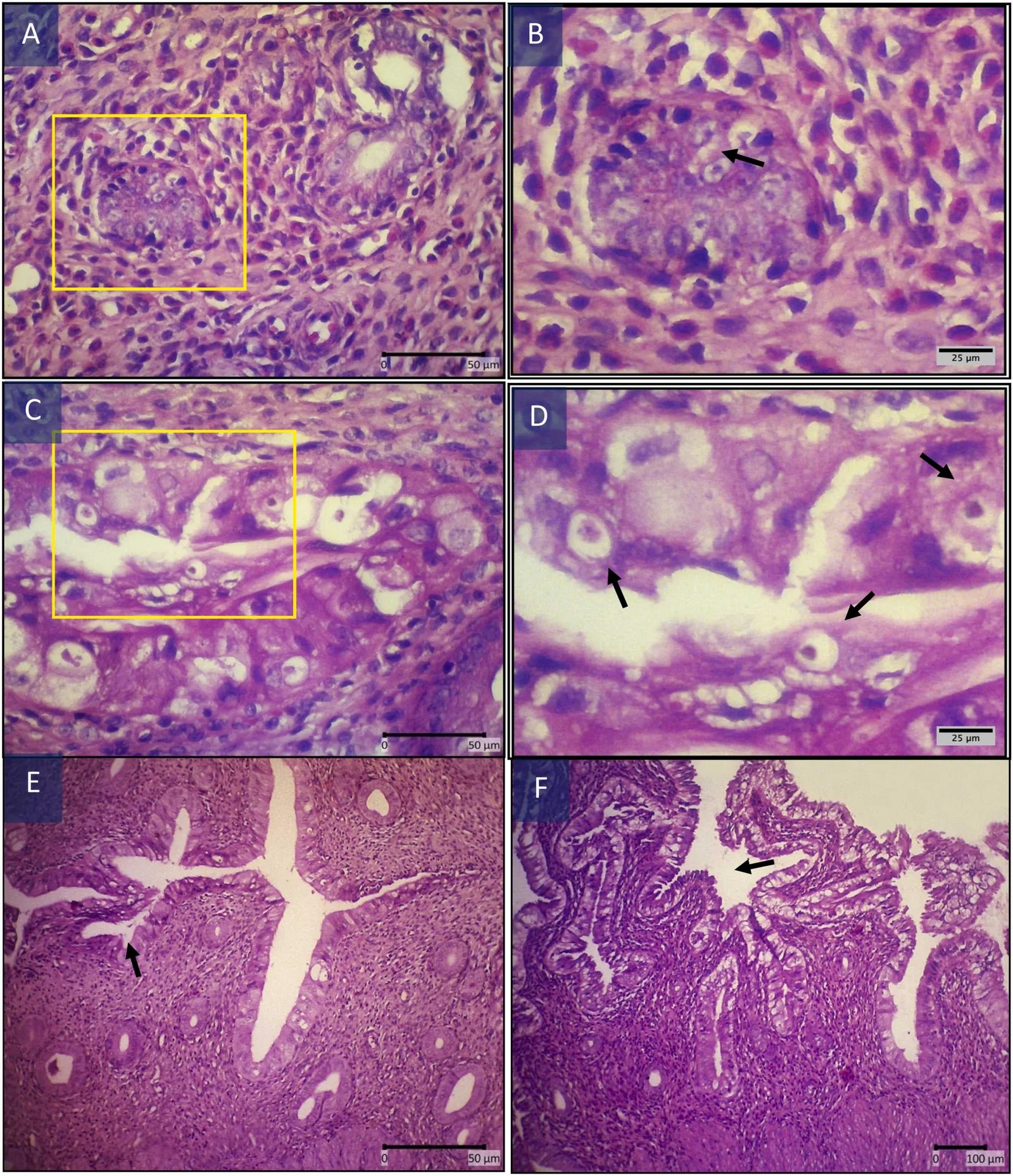
Endometrial glands in the DES-treated group. (A) Marked proliferation of atypical endometrial epithelial cells is observed, with evident nuclear polarity. (B) Higher magnification of A. (C) A heterogeneous population of atypical endometrial epithelial cells exhibiting nuclear polarity, along with the presence of clear cells. (D) Higher magnification of panel C. (E) EC exhibiting a villoglandular growth pattern, showing a papillary growth pattern of intermediate grade, (F) with mucinous epithelium. (Scale bar 50 µm; original magnification ×400 using a 40 × objective; inset ×1000; Nikon Eclipse Ci-E microscope; Nikon DS-Fi2 digital camera; Nikon Image System software.).

Immunohistochemical staining revealed ERα expression in uterine epithelial and endometrial glandular cells (Fig. 8).

**Fig. 8:**
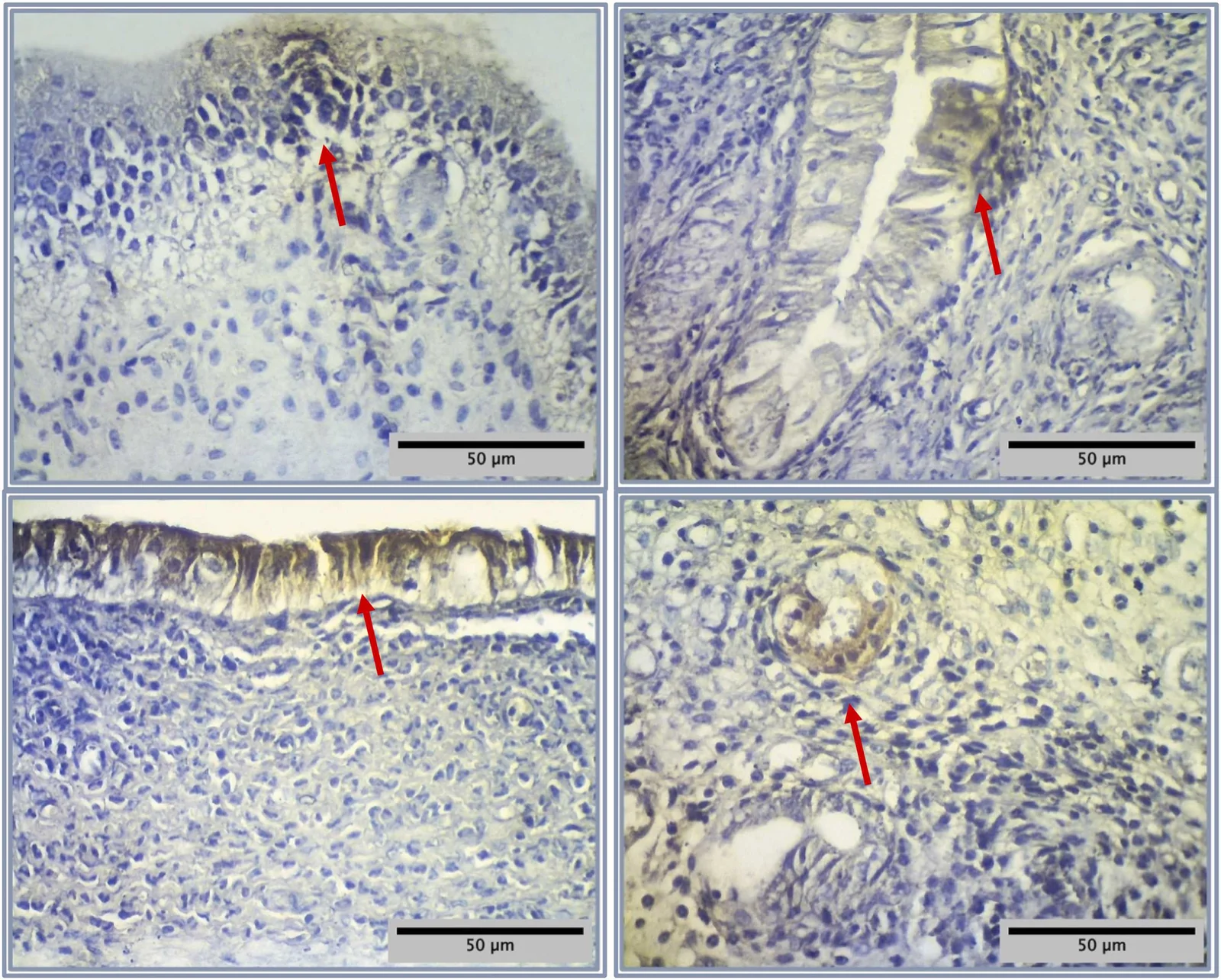
ERα expression was observed in uterine epithelial cells and endometrial glandular cells, as demonstrated by immunohistochemical staining. (Scale bar: 50 µm; Objective lens 40 ×; Eclipse E-i microscope; DS-Fi2 camera, 300 megapixels.).

## Discussion

The present study demonstrates that DES-induced endometrial carcinogenesis is characterized by dynamic and non-linear modulation of ERα expression, which closely parallels histopathological tumor progression and aggressiveness with worsening histopathological grade. By integrating morphological features with ER immunohistochemistry, our findings provide insight into the temporal evolution of estrogen dependence during endometrial tumorigenesis. These observations are consistent with the classical pathogenetic model of estrogen-driven endometrial carcinogenesis, in which prolonged estrogenic stimulation initiates tumor development, followed by molecular divergence during progression [11], [12].

### Uterine ERα expression is associated with the development of EC and histopathological grade in mice exposed to DES

Diethylstilbestrol exposure is associated with a significant increase in ERα expression (Fig. 2, Fig. 3) across groups and that ERα upregulation aligns with worsening histopathological grade (Fig. 6, Fig. 7), with the most severe lesions concentrated in the higher expression groups (notably P2.2 and P3.2) These patterns support an estrogen-driven model of endometrial carcinogenesis in which sustained receptor activation promotes proliferative and dysplastic remodeling of endometrial epithelium. Mechanistically, ERα is a central transcriptional regulator in EC biology, promoting cell cycle progression, survival, invasion, and anti-apoptotic signaling through classical E2/ERα transcriptional programs and context dependent coregulator activity [13], [14].

Our path analysis further strengthens biological plausibility by showing associations between DES and ERα expression, as well as a downstream effect of ERα on EC outcome (Fig. 5), consistent with prior experimental evidence that the adverse reproductive tract consequences of neonatal DES exposure are ERα-dependent. In murine systems, ERα has been shown to mediate critical aspects of DES-driven developmental toxicity within reproductive tissues, indicating that receptor engagement is not merely correlative but functionally required for key downstream phenotypes [15]. This ERα dependence is consistent with the broader concept that synthetic estrogen exposure can “program” uterine responsiveness and tissue fate, thereby creating a pro-neoplastic milieu that manifests later as pathological progression.

To further explain the underlying biological mechanisms, DES-induced carcinogenesis can be understood through both genomic and non-genomic estrogen signaling pathways. DES is a synthetic nonsteroidal estrogen that exerts carcinogenic effects through prolonged estrogenic stimulation in the absence of progesterone counterbalance (unopposed estrogen), leading to atypical endometrial hyperplasia and eventual malignant transformation. High-dose DES administration increases estrogen levels, which interact genomically by binding to estrogen receptors, followed by receptor dimerization and translocation from the cytoplasm to the nucleus. In the nucleus, estrogen binds to estrogen response elements (EREs) and functions as a transcription factor, promoting mitogenic signaling, DNA damage, and epigenetic alterations that contribute to carcinogenesis. This estrogenic stimulation induces mitogenic activity, resulting in malignant transformation of endometrial glandular epithelium into endometrial carcinoma [16]. DES has also been shown to modulate the Wnt/β-catenin signaling pathway, leading to overexpression of proliferation markers such as Ki-67 [6].

In addition to genomic mechanisms, estrogen also exerts non-genomic effects by binding to estrogen receptors located on the plasma membrane, which subsequently interact with receptor tyrosine kinases (RTKs) such as insulin-like growth factor receptor 1 (IGF-1R) and ErbB2/HER2, activating downstream signaling pathways including phosphoinositide 3-kinase (PI3K) and mitogen-activated protein kinase (MAPK) [17]. Activation of the PI3K pathway stimulates AKT and mTOR signaling, promoting cell survival, while ErbB2/HER2 activation enhances the Ras/Raf/MAPK pathway, leading to increased expression of oncogenic mediators such as MYC, BCL-2, AXIN2, and Cyclin D1. These pathways collectively promote cellular proliferation and increase Ki-67 expression, thereby contributing to endometrial carcinogenesis [18].

A plausible mechanistic bridge between early estrogenic exposure and later neoplastic risk is persistent reprogramming of gene expression through epigenetic remodeling. Developmental exposure to endocrine active compounds including DES has been linked to enduring DNA methylation and transcriptomic alterations in adult tissues, with ERα implicated in mediating these long range molecular effects [19], [20]. More broadly, contemporary reviews of endocrine disrupting chemicals emphasize epigenetic dysregulation as a key pathway by which estrogenic exposures yield durable changes in gene regulatory networks relevant to reproductive tract disease [11]. The present results ERα elevation accompanied by higher grade histopathology are consistent with a model in which DES enhances ER signaling capacity, which may then cooperate with epigenetic and inflammatory/stromal remodeling processes to accelerate glandular atypia and malignant transformation.

Recent mechanistic work also highlights that uterine epithelial identity is maintained by a balance of signaling programs that can be disrupted by potent estrogens. Genetic evidence indicates that retinoic acid (RA) signaling antagonizes estrogen signaling to maintain adult uterine cell fate, and neonatal exposure to DES perturbs this balance, contributing to loss of normal epithelial homeostasis [21]. This framework provides a contemporary explanation for why sustained estrogenic activation may translate into architectural disruption and higher histologic grade: once homeostatic “brakes” on estrogen signaling are weakened, endometrial epithelium may become increasingly permissive to hyperplastic and dysplastic remodeling.

Importantly, the relationship between ERα and tumor aggressiveness can be context dependent. While ERα is commonly oncogenic in estrogen responsive EC biology, ERα expression may be retained in well-differentiated disease and can decline as tumors dedifferentiate [14]. This nuance is relevant to interpretation: our graded histopathology and ERα pattern may reflect an estrogen dependent trajectory where ERα upregulation contributes to lesion initiation and progression within an experimental window, rather than late stage dedifferentiation where ERα loss may emerge. Positioning the findings in this temporal framework strengthens biological coherence with current EC literature [13], [14]. In clinical datasets, ERα signaling also remains prognostically meaningful; for example, ERα overexpression has been associated with worse outcomes in endometrioid EC, underscoring the clinical relevance of heightened estrogen signaling in at least a subset of cases [22].

Collectively, these findings support a model in which DES acts as a potent estrogenic initiator of endometrial carcinogenesis through ER-mediated mechanisms, while prolonged exposure promotes molecular reprogramming and ER downregulation, facilitating tumor progression and invasiveness. This dual role of estrogen as both initiator and indirect promoter of hormone independent growth has been proposed in experimental models of endocrine related carcinogenesis and aligns with long term observations in DES exposed populations [23], [24].

This study has limitation that should be considered. Intra- and inter-observer variability were not formally assessed, which may introduce potential measurement bias. However, to minimize variability, all evaluations were conducted using standardized protocols by two independent experienced investigators. In addition, future studies with larger sample sizes and formal reproducibility assessments are warranted to validate these results.

Finally, these experimental findings should be interpreted alongside the established human evidence base on DES. Public health and epidemiologic resources recognize DES as a potent endocrine disruptor associated with specific long term adverse outcomes in exposed females, including elevated risk of clear cell adenocarcinoma of the lower genital tract and increased risks of several reproductive tract abnormalities [25], [26]. Although population level cancer risk patterns depend on exposure timing, dose, and outcome definition, the convergence of mechanistic (ERα-dependent programming; epigenetic remodeling; disrupted uterine homeostasis) and phenotypic (higher grade pathology with higher ERα) evidence supports the translational conclusion that ER signaling is a key node linking DES exposure to endometrial disease progression. Taken together, our results justify further work integrating ERα pathway readouts with additional oncogenic axes (e.g., PI3K/AKT/PTEN, inflammatory signaling, stromal-epithelial crosstalk) and expanding sample size to refine effect estimates and dose response structure.

## Conclusion

DES is associated with endometrial carcinogenesis through ERα-dependent mechanisms in vivo. Increased ERα expression parallels histopathological progression, and mechanistic analysis suggests ERα as a key mediator linking synthetic estrogen exposure to tumor development. These findings underscore the central role of estrogen signaling in DES induced EC.

## CRediT authorship contribution statement

**Widjiati Widjiati:** Validation, Supervision, Resources, Investigation, Formal analysis, Data curation. **Peng-Hui Wang:** Writing – review & editing, Validation, Supervision, Methodology, Investigation, Formal analysis, Data curation. **Brahmana Askandar Tjopkroprawiro:** Writing – review & editing, Visualization, Validation, Supervision, Methodology. **Setyo Teguh Waluyo:** Writing – review & editing, Writing – original draft, Visualization, Validation, Software, Resources, Project administration, Methodology, Investigation, Funding acquisition, Formal analysis, Data curation, Conceptualization.

## Declaration of Competing Interest

The authors declare that they have no known competing financial interests or personal relationships that could have appeared to influence the work reported in this paper
